# Supplementary material for: Learning curve and surgical outcome of robotic assisted colorectal surgery with ERAS program
Source: Sci Rep. 2022 Nov 29;12:20566. doi: 10.1038/s41598-022-24665-w (PMC9709162; doi:10.1038/s41598-022-24665-w)
Supplement: Supplementary file 1 — Supplementary Information 1. [file 41598_2022_24665_MOESM1_ESM.zip › LSC Raw data-Ñ[▒K/145/H_ñw╝╨░O▒Kñσ.pdf]

正式摘要(電子簽章：000000231697122)

W52 / W52 ☐ 英文標題(注意:無英文姓名-請補病人基本檔!) ☐【讀取】憑證

臺中榮民總醫院

出院病歷摘要

轉入號： 轉出號：

[redacted]

(8)流水編號	
(9)入院日期	110/11/28 (2021) 直腸外科 W52 - 082
(10)轉科日期	
(11)出院日期	110/12/07 (2021) 住院天數計 9 日

診斷

- 入院：1. Rectal tumor, 10cm FAV
  2. HTN, without medication control
  3. Acute appendicitis s/p OP in 2005
  4. Left inguinal hernia s/p OP in 2006
- 出院：1. Locally advanced S-colon with seeding status, s/p Laparoscopic AR on 2021/11/29
  2. HTN, without medication control
  3. Acute appendicitis s/p OP in 2005
  4. Left inguinal hernia s/p OP in 2006

主訴

- intermittent bloody stool since 2021/03

病史

- This is a 83 years old male patient with history of

  1. HTN, without medication control

  2. Acute appendicitis s/p OP in 2005

  3. Left inguinal hernia s/p OP in 2006

  According to the patient, he had intermittent bloody stool since 2021/03, and the symptoms progress in recent days, so he visit LMD on 11/18 and then refer to our CRS OPD, where colonoscopy was arranged and showed rectal tumor, 10cm FAV with partial obstruction. LAb data showed CEA: 55.37. Abdominal CT showed no obvious distant metastasis. Under the impression of rectum cancer, this time he was admitted to our ward for opeartion.

體檢發現

-
  - General appearance :
    - easy looking , conscious clear , without cardiopulmonary distress
    - Vital signs自動帶入date 體溫(BT) 脈搏(P) 呼吸(R) 血壓(BP) 身高(BH) 體重(BW) BMI
      20211128 35.8℃ 58次/分 18次/分 152/71mmHg 170.4cm 69.1kg 23.8
    - Vital signs: B.P. 152/71 mmHg , P.R. 58 /min , R.R. 18 /min , B.T. 35.8 ℃
  - HEENT :
    - Eyes: no anemic conjunctiva, no icteric sclera, normal EOM
    - ENT: no otorrhea, no rhinorrhea, no erythematous mucosa, no injected tonsil, no deformity
  - Neck: supple, freely movable, trachea midline,no deformity, no engorged jugular vein , no palpable thyroid or lymph node enlargement
  - Chest:
    - Inspection: Grossly normal, symmetric and fully expansion, no accessory muscle used, no operation scar, no spider angiomata, no gynecomastia
    - Palpation: no palpable mass or nodule, no subcutaneous emphysema, no tenderness
    - Ausculation: clear breathing sound
  - Heart:
    - Palpation: no thrill, no heave
    - Percussion: normal heart size
    - Ausculation: regular heart beat, normal S1, S2, no gallop, no murmur
  - Extremities: No deformity, full ranged of motion without limitation, no clubbing finger or nail cyanosis, no pitting edema, no ulcer or pigmentation, no obvious skin lesion, no varicose vein,

- no bone, joint or muscle tenderness

CRS CONDITION

- Karnofsky performance status: (100) Normal, no complaints, no evidence of disease
- PE of Abdomen:
- CEA: 55.37 ng/ml
- Imaging Studies:
- Colonoscope report: .
- Figures:
  1. colon & rectum

手術日期及方法

- 手術日期及方法20211129 1. Laparoscopic Anterior resection and anastomosis
  2. Intraoperative colonoscopy : (-)
  3. High ligation (+) : At IMA root. Left colic artery and IMV were also ligated
  4. Take down from the splenic flexure : (-)
  5. EndoGIA (60-4.1) X2 + EEA (29 mm) were used
  6. Proximal cut end: 13cm, distal cut end: 3 cm
  7. One 12 Fr. penrose drain was placed into the pelvic area from LLQ trochar site

住院治療經過

- After admission, he received Laparoscopic Anterior Resection on 2021/11/29. We keep him NPO with NG decompression after OP, and removed NG tube on 12/02. We let him start try water since 12/03. Due to no specific discomfort, we let him try bland I diet on 12/04. Follow up lab data on 12/06 showed neutropenia. We gave him GCSF and follow up lab on 11/07. Lab data on 11/07 showed Leukocytosis, we suggest Consult HEMA, but the patient's family claimed that the patient had similar episode before and ever visit HEMA doctor at other hospital, but no specific diagnosis. After discussion, they decided to follow up HEMA at Tainan and the patient was discharged on 12/07 and arrange CRS OPD follow up and suggest HEMA follow.

合併症

一般檢查

- 累積報告 :

DATE	TIME	ALB	TP	BIL.T	BIL.D	ALKP	AST	ALT	LDH	AMY	LIPASE	NH3	Ferritin	UA
1101129	1927	3.0		0.5				13						
1101202	0907	2.8		0.5				8						
1101206	0904	3.3		0.4	0.1	69	19	6						
1101207	0852			0.53				8						

DATE	TIME	WBC	RBC	HGB	HCT	MCV	PLT	BLST	NEUT	BAND	SEG	LYM	MON	EOS	BAS
1101129	1928	5850		11.3	34.2		81			1.0	86.0	10.0	3.0	0.0	0.0
1101202	0849	3080	3.92	11.4			73		66.9			16.6	16.2	0.3	0.0
1101206	0858	1450	3.93	11.7	35.4	90.1	103				42.0	28.0	28.0	2.0	0.0
1101207	0857	35390	4.15	12.0	37.0	89.2	119			1.0	94.0	1.0	4.0	0.0	0.0

DATE	TIME	NA	K	CL	BUN	CREAT	eGFR	CA	Mg	P	CRP	ESR	PCT	D-Dimer	LACTATE
1101129	1927	142	3.4		14	0.83		7.1							
1101202	0907	139	3.6	110	5	0.81	96.73	6.8							
1101206	0730														10.0
1101206	0904	141	4.2		11	0.84	92.75	7.4			1.108				
1101207	0733														16.1
1101207	0852	140	3.9		13	0.89	86.76								
1101207	0855												0.127		

DATE	TIME	SP-GR	PH	PROT	GLUC	KETON	BILI	UROB	OB	NITR	LEU	RBC	WBC	EPCEL
1101207	1222	1.017	5.5	Negative	Negative	Negative	Negative	0.2	Negative	Negative	Negative	0-2	2-5	0-1

特殊檢查

病理報告

其它

出院時情況

- 3.改門診治療

出院指示

-

藥名	劑量	劑量單位	用法	頻次	數量	數量單位	現況	天數	單位
Ultracet tab	1	TAB	PO	BIDPRN	14	TAB	TH	7	天

出院計劃

- 門診日期
    - date on 2021/12/15

主治醫師 2062F 蔣鋒帆 / 住院醫師 2339C 蔡濬樟

正式摘要(電子簽章：**000000231697122)**

W52 / W52

doctorRank:B
